# Integrative Single-Cell and Bulk Transcriptomic Analysis Identifies Macrophage-Related Gene Signatures Predictive of Hepatocellular Carcinoma in Cirrhosis 

**DOI:** 10.3390/genes16101213

**Published:** 2025-10-15

**Authors:** Zhongyuan Zhang, Chuisheng Zeng, Xuetong Yong, Wenping Zhou, Yongfang Xie, Jianzhong Shu

**Affiliations:** 1 Chongqing Key Laboratory of Big Data for Bio Intelligence, Chongqing University of Posts and Telecommunications, Chongqing 400065, China; 19823318477@163.com (Z.Z.);; 2 Chongqing University of Traditional Chinese Medicine, Chongqing 402760, China; 3 Chongqing Traditional Chinese Medicine Hospital, Chongqing 400020, China

**Keywords:** single-cell RNA sequencing, liver cirrhosis, hdWGCNA, machine learning, predictive model

## Abstract

**Background/Objectives**: Liver cirrhosis is a major global health challenge and a key risk factor for hepatocellular carcinoma (HCC), a malignancy with high mortality due to late diagnosis. This study aimed to integrate single-cell RNA sequencing (scRNA-seq) and bulk RNA sequencing (bulk RNA-seq) data, using single-cell data to identify macrophage-associated transcriptomic changes during the progression from cirrhosis to HCC, and using bulk data to validate these findings in independent cohorts, while developing predictive models for early risk assessment. **Methods**: We integrated single-cell RNA sequencing (scRNA-seq) and bulk RNA sequencing datasets derived from liver tissues of cirrhosis and HCC patients. Single-cell data were used to identify macrophage subtypes and their dynamic transcriptional changes, while bulk data provided validation in independent cohorts. Gene expression and network analyses were performed, and candidate genes were used to construct diagnostic models with Lasso regression, Random Forest, and Extreme Gradient Boosting (XGBoost). Model performance was evaluated using receiver operating characteristic curves. **Results**: We identified eleven macrophage-associated genes, among which KLK11, MARCO, CFP, KRT19, GAS1, SOD3, and CYP2C8 were downregulated in HCC, indicating loss of tumor-suppressive and pro-apoptotic functions, while TOP2A, CENPF, MKI67, and NUPR1 were upregulated, reflecting enhanced cell cycle progression, proliferation, and M2 polarization. These are all associated with the progression from liver cirrhosis to HCC. Based on these findings, we established predictive models using Lasso, Random Forest, and XGBoost, which stratified cirrhotic patients into high- and low-risk groups according to cutoff values using liver tissue transcriptomic data. All three models demonstrated high diagnostic performance. **Conclusions**: This study highlights the critical role of macrophage-associated transcriptomic remodeling in liver disease progression. The machine learning–based predictive models offer a promising approach for early diagnosis and clinical decision-making in patients with cirrhosis.

## 1. Introduction

HCC is a classic example of an inflammation-associated cancer, with over 90% of cases developing in the context of chronic liver injury [1]. Risk factors, such as cirrhosis, trigger persistent inflammatory responses characterized by infiltration of macrophages and immature myeloid cells, as well as dysregulated cytokine production [2,3,4,5]. These unresolved responses drive progressive fibrosis, cirrhosis, and ultimately, HCC [6,7,8].

Hepatic macrophages play a central role in liver homeostasis and disease, participating in diverse processes including injury exacerbation, anti-inflammation, tissue repair, regulation of fibrosis (both pro- and anti-fibrotic roles), and tumor progression (both pro- and anti-tumorigenic functions) [9]. Tumor-associated macrophages (TAMs), one of the most abundant immune cell populations within the tumor microenvironment, significantly contribute to HCC progression through inflammation-mediated mechanisms. Based on their origin, TAMs can be classified as either tissue-resident macrophages, such as Kupffer cells (KCs), or monocyte-derived macrophages (MoMφs) [10,11]. Under physiological conditions, KCs are the most common. However, during HCC development, both KCs and MoMφs undergo phenotypic changes driven by tumor-promoting signals, ultimately contributing to immune remodeling and tumor growth [12].

With the rapid advancement of high-throughput sequencing technologies and artificial intelligence, computational methods have emerged as powerful tools for dissecting the molecular mechanisms driving the progression from cirrhosis to HCC [13,14]. In recent years, machine learning (ML) has gained momentum in oncology, offering solutions to challenges posed by cancer’s complexity and data dimensionality [15]. These techniques facilitate early diagnosis, risk stratification, and personalized treatment planning, thereby improving clinical outcomes [16,17].

Among commonly used ML algorithms, random forest and extreme gradient boosting (XGBoost) are ensemble-based approaches that perform well on structured biological data [18,19,20]. Lasso regression is another effective method for performing variable selection and regularization and is especially suited for high-dimensional transcriptomic datasets.

In this study, we aim to explore the dynamic changes that occur in hepatic macrophages during the progression from cirrhosis to HCC by integrating single-cell transcriptomic and bulk RNA sequencing data. We further utilize ML algorithms—Lasso regression, random forest, and XGBoost—to construct a predictive model based on macrophage-associated gene expression features. Our objective is to develop a robust diagnostic tool with which to identify cirrhotic patients who are at high risk of developing HCC, thereby facilitating early intervention and improving clinical outcomes.

## 2. Materials and Methods

### 2.1. Data Collection

scRNA-seq datasets were obtained from GEO, including GSE136103, GSE186343, GSE242889, and GSE202642. These datasets comprise healthy, cirrhotic, and HCC liver tissues, all generated using the 10× Genomics Chromium platform with sequencing on Illumina HiSeq or NovaSeq instruments. For downstream analysis, we used the processed gene–cell count matrices provided by GEO, while raw FASTQ files are available in the SRA.

Bulk RNA-seq datasets, GSE25097 and GSE14323, were likewise retrieved from GEO. GSE25097 (Affymetrix Rosetta/Merck Human RSTA 1.0 array, GPL10687) contains 268 HCC, 243 adjacent non-tumor, 40 cirrhosis, and 6 normal samples, while GSE14323 (Affymetrix U133A/U133A 2.0 arrays, GPL96/GPL571) comprises 124 samples including HCV-related HCC, cirrhosis, and normal liver tissues. In both datasets, normalized expression matrices provided by GEO were used for analysis, with raw CEL files also accessible.

### 2.2. Data Preprocessing and Analysis for Single-Cell RNA Sequencing

In this study, scRNA-seq data were processed using the R package Seurat (version 5.1.0) (https://www.github.com/satijalab/seurat) (accessed on 30 September 2024) [21]. Seurat objects were initially created in bulk using the CreateSeuratObject function. Quality control procedures were then applied to filter out low-quality cells based on metrics such as gene count, unique molecular identifiers (UMIs), and mitochondrial gene expression.

Following quality control, data normalization and identification of highly variable genes were performed using the SCTransform method according to Seurat’s official guidelines [22]. To further mitigate batch effects across multiple datasets, we applied Reciprocal PCA (RPCA)–based integration implemented in Seurat’s IntegrateLayers function, combined with SCT normalization. This approach identifies shared anchors across datasets, aligns them into a common expression space, and effectively corrects technical variability while preserving true biological signals, thereby enhancing the consistency and reproducibility of downstream analyses.

Next, principal component analysis (PCA) was conducted using the RunPCA function for linear dimensionality reduction. Based on the inflection point observed in the ElbowPlot, the top 20 principal components were selected for subsequent analyses. Dataset integration was performed following Seurat’s standard workflow to correct for inter-sample batch effects.

After integration, cell clustering was carried out using the FindClusters function, with a resolution parameter set to 0.5. Clustering was based on the construction of a shared nearest neighbor (SNN) graph. To visualize the spatial distribution of cell clusters in two dimensions, Uniform Manifold Approximation and Projection (UMAP), a nonlinear dimensionality reduction algorithm, was applied [23].

Differentially expressed genes (DEGs) for each cluster were identified using the FindAllMarkers function. Cell type annotation was performed by referencing the CellMarker 2.0 database (http://117.50.127.228/CellMarker) (accessed on 30 October 2024) [24] and the Cell Taxonomy database (https://ngdc.cncb.ac.cn/celltaxonomy/) (accessed on 30 October 2024) [25], and by examining the expression profiles of canonical marker genes for specific cell populations.

### 2.3. Differential Expression Analysis and Gene Set Enrichment Analysis

Differential expression analysis was conducted to identify genes with significantly altered expression between different disease stages. Specifically, macrophage-labeled cells were extracted, and the FindMarkers function in Seurat was applied to identify differentially expressed genes (DEGs) between cirrhosis and HCC.

To explore the biological roles of macrophage-related DEGs, GSEA was performed. Functional enrichment was carried out using the clusterProfiler package in R [26,27]. Gene Ontology (GO) annotations were used to categorize enriched functions into three domains: biological process (BP), molecular function (MF), and cellular component (CC).

### 2.4. Cell Interaction Analysis

To elucidate the complex landscape of cell–cell communication within the tumor microenvironment, we utilized the CellChat package, which enables quantitative analysis of interaction networks based on ligand–receptor–cofactor databases derived from scRNA-seq data [28]. Following the standard CellChat workflow, we systematically quantified and characterized the dynamics, strength, and signaling pathways of intercellular communication. Particular emphasis was placed on comparing macrophage-mediated interactions between cirrhosis and HCC to better understand their functional heterogeneity under distinct pathological conditions [29].

### 2.5. High-Dimensional Weighted Gene Co-Expression Network Analysis (hdWGCNA)

To identify genes closely associated with disease-specific macrophage subclusters, we applied high-dimensional weighted gene co-expression network analysis (hdWGCNA) to the macrophage scRNA-seq data. Initially, macrophage-labeled cells were extracted, re-normalized, and highly variable genes were re-identified. Dimensionality reduction and nonlinear dimensionality reduction were then performed to characterize cell subclusters.

Next, hdWGCNA was conducted on the expression profiles of these subclusters. For metacell construction, the parameter k was set to 12, and a soft threshold power of 8 was chosen based on data characteristics to build the co-expression network. This analysis identified 13 highly correlated gene modules. For each module, the top 20 genes with the highest module membership scores were defined as hub genes.

To pinpoint modules related to macrophage-specific transcriptional changes, differentially expressed genes (DEGs) identified in macrophages were intersected with genes in each module. Modules in which more than 10% of their hub genes overlapped with macrophage DEGs were considered differentially regulated modules.

### 2.6. Transcriptomic Data Analysis and Gene Selection

In this study, transcriptomic data from the GSE25097 dataset were processed using the R package affy to import robust multi-array average (RMA) expression data and convert them into a standardized gene expression matrix. Differential expression analysis was then performed using the limma package to identify significantly dysregulated genes. To enhance biological relevance, the differentially expressed genes identified from the bulk RNA-seq data were intersected with both the macrophage-specific differentially expressed genes and the module-related genes derived from the single-cell transcriptomic analysis. The overlapping genes were selected as candidate biomarkers for downstream model construction. To further refine the gene set and develop a robust diagnostic model, least absolute shrinkage and selection operator (LASSO) regression analysis was applied to the GSE25097 dataset using the R package glmnet. The penalty parameter was optimized by cross-validation, and a lambda value of 0.0004814197 was selected to construct the final LASSO regression model comprising the most informative genes.

### 2.7. Model Construction and Performance Evaluation

To develop diagnostic models for cirrhosis and HCC, three machine learning approaches were employed: random forest, extreme gradient boosting (XGBoost), and least absolute shrinkage and selection operator (LASSO) regression. The random forest model was constructed using gene expression profiles from the GSE25097 dataset as the training set and those from the GSE14323 dataset as the validation set. A total of 500 decision trees were used during model training, and feature importance and proximity metrics were enabled. The receiver operating characteristic (ROC) curve was plotted, and the area under the curve (AUC) was calculated to assess model performance. Subsequently, the training and validation sets were combined to form an integrated dataset, during which platform-related batch effects were adjusted using the removeBatchEffect function in the limma package, thereby ensuring data comparability before further evaluation of model robustness and generalizability.

For the XGBoost model, a binary logistic regression objective was used. Key parameters included a learning rate of 0.3, a maximum tree depth of 6, a log loss evaluation metric, and 100 training rounds. Following prediction on the validation dataset, the ROC curve and corresponding AUC were computed to determine diagnostic accuracy. The LASSO regression model, trained on the same datasets, was evaluated using an identical strategy. All three models were implemented for the classification of cirrhosis and HCC. For each model, the optimal cutoff value was determined by selecting the threshold that yielded the best diagnostic performance based on ROC curve analysis, considering the highest sensitivity and specificity. Patients were subsequently stratified into high-risk and low-risk groups according to this cutoff. The three models were then jointly applied to establish predictive frameworks for early diagnosis.

## 3. Results

### 3.1. Single-Cell Transcriptomic Landscape of Hepatitis, Cirrhosis, Hepatocellular Carcinoma, and Adjacent Non-Tumorous Liver Tissue

**Figure 1:**
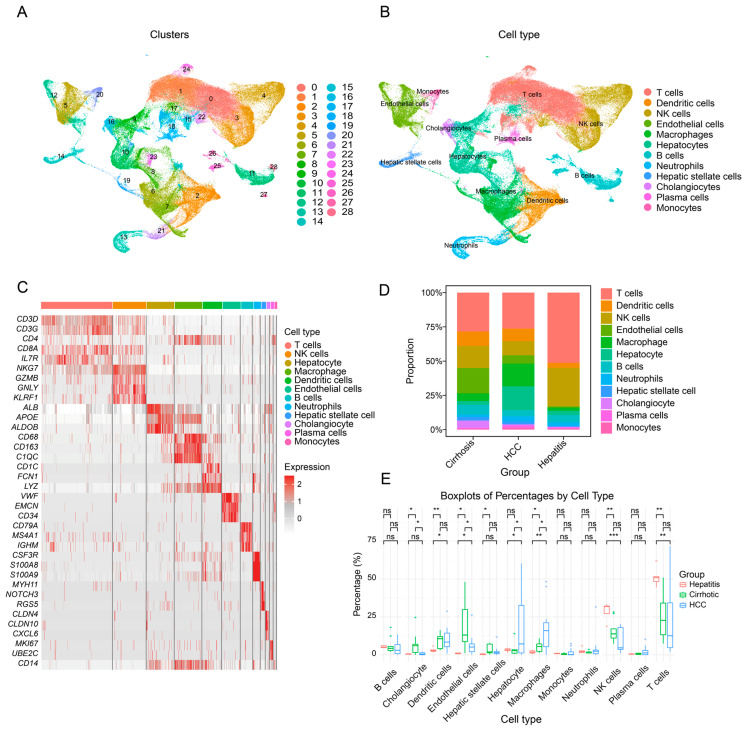
Single-cell transcriptomic landscape and cellular composition of liver disease samples. (**A**) UMAP plot showing the distribution of samples following the removal of batch effects. (**B**) UMAP plot displaying different cell types in liver disease. (**C**) Heatmap showing the expression of representative marker genes across 12 major cell types. (**D**) Stacked bar plot comparing the relative proportions of each cell type across different disease stages (hepatitis, cirrhosis, and HCC). (**E**) Boxplot showing the proportion differences in multiple cell types across different liver disease states, with macrophages exhibiting a progressive. (Statistical significance is indicated as follows: ns, not significant; * *p* < 0.05; ** *p* < 0.01; *** *p* < 0.001).

We processed and integrated single-cell RNA sequencing (scRNA-seq) data from four publicly available datasets (GSE136103, GSE186343, GSE242889, and GSE202642), yielding a total of 124,148 cells. Among these, 25,337 cells were derived from liver cirrhosis samples, 20,928 from hepatitis samples, and 77,883 from HCC samples. After standard normalization, clustering, integration, and nonlinear dimensionality reduction, we identified 29 distinct cellular clusters (Figure 1A). By referencing public databases and evaluating the expression of canonical marker genes, these clusters were preliminarily annotated into 12 major cell types: T cells (CD3D, IL7R, CD69, NKG7, GZMA, CD2, CD8A, CD3G, CD4, TRAC, FOXP3, TRDC), natural killer (NK) cells (NKG7, GZMB, GNLY, PRF1, KLRF1), macrophages (CD14, CD68, CD163, CTSD, ADAP2, C1QC), dendritic cells (CD1C, CSTA, FCN1, LYZ), endothelial cells (VWF, EMCN, CD34, VWA1, PODXL), B cells (CD79A, MS4A1, MZB1, IGKC, IGHM), neutrophils (CSF3R, S100A8, S100A9, MNDA), hepatocytes (ALB, APOC2, APOE, ALDOB, APOC1), hepatic stellate cells (MYH11, NOTCH3, RGS5), cholangiocytes (CLDN4, CLDN10, CXCL6), plasma cells (MKI67, UBE2C), and monocytes (CD14) [24,25]. The final cell count for each type was as follows: T cells (38,460), dendritic cells (10,464), NK cells (18,000), endothelial cells (9756), macrophages (14,745), hepatocytes (14,901), B cells (6497), neutrophils (3786), hepatic stellate cells (2461), cholangiocytes (1976), plasma cells (1677), and monocytes (1425) (Figure 1B,C). These quantifications provided a foundational dataset for subsequent analyses, ensuring the accuracy and reliability of downstream investigations (Figure S1, Table S1). We further compared the relative proportions of these cell types across different disease stages. (Figure 1D). The results revealed significant compositional changes in many cell populations during disease progression. Notably, the proportion of macrophages increased progressively from hepatitis to cirrhosis and ultimately to HCC, with statistically significant differences (*p* < 0.05) (Figure 1E). Considering that cirrhosis is a primary precursor of HCC and that changes in cellular and microenvironmental composition between these two stages hold critical clinical and biological implications, we focused subsequent analyses specifically on cirrhosis and HCC. In particular, we aimed to elucidate the dynamic states and functional diversity of macrophages during the transition from cirrhosis to HCC.

### 3.2. Macrophage Profiling in Hepatic Diseases: Gene Expression, Pathways, and Polarization Dynamics

**Figure 2:**
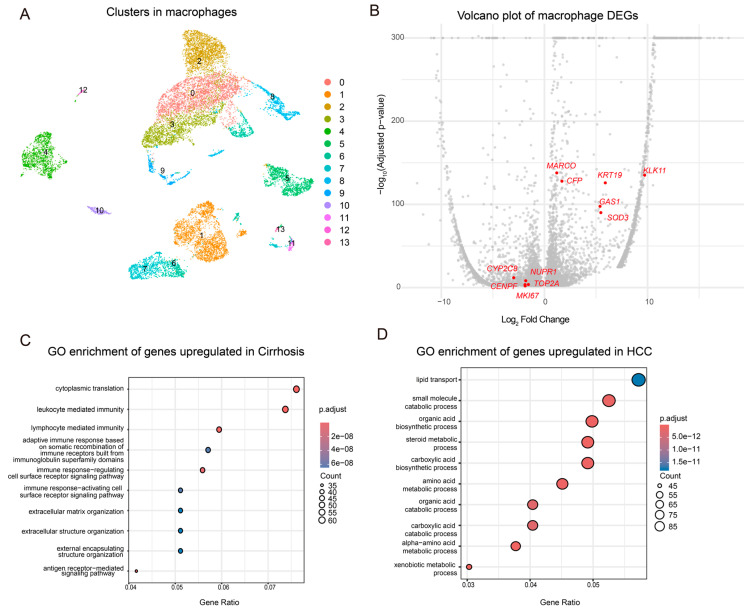
Differential gene enrichment and macrophage polarization dynamics during the transition from cirrhosis to HCC. (**A**) UMAP plot showing reclustered macrophages extracted from all cell populations. Each color represents a distinct macrophage subcluster identified by unsupervised clustering. (**B**) Volcano plot of DEGs in macrophages between cirrhosis and HCC, Eleven key DEGs with potential diagnostic or functional relevance (MARCO, KLK11, CFP, KRT19, GAS1, SOD3, CYP2C8, NUPR1, TOP2A, CENPF, MKI67) are highlighted. (**C**) Gene ontology and pathway enrichment analysis of DEGs upregulated in cirrhosis-associated macrophages. (**D**) Gene ontology and pathway enrichment analysis of DEGs upregulated in tumor-associated macrophages.

First, we extracted all cells annotated as macrophages from the integrated single-cell dataset (Figure 2A). Subsequently, we identified a total of 6408 DEGs in macrophages between the stages of cirrhosis and HCC. After applying stringent filtering criteria (*p*_val_adj < 0.01; pct.1 > 0.25), 4144 high-confidence DEGs were retained for downstream analyses (Figure 2B). These were categorized into upregulated and downregulated genes in cirrhosis, and each group was subjected to gene enrichment analysis to elucidate biological pathways associated with their differential expression. The pathways enriched in cirrhosis-associated macrophages revealed prominent immune activation and inflammatory responses, including leukocyte-mediated immunity, lymphocyte activation, adaptive immune pathways, chemotaxis, and immunoregulatory signaling. These findings highlight the pivotal role of macrophages in promoting liver fibrosis and modulating the immune response: by enhancing antigen presentation, migration, and chemotactic activity, macrophages facilitate the recruitment and activation of inflammatory cells to damaged hepatic tissues, thereby sustaining and exacerbating chronic inflammation. Furthermore, their active involvement in organization and remodeling of the extracellular matrix contributes to the progression of fibrosis. The upregulation of protein synthesis-related pathways reflects the highly activated state of these macrophages, marked by sustained secretion of pro-inflammatory cytokines and immune modulators (Figure 2C).

In contrast, tumor-associated macrophages in HCC exhibited notable metabolic reprogramming, characterized by increased expression in pathways related to xenobiotic metabolism, lipid metabolism, and amino acid metabolism. This metabolic shift reflects the role of macrophages in adapting to the tumor microenvironment, regulating immune responses, and supporting tumor progression. Such differences underscore the heterogeneity of macrophages between the stages of cirrhosis and HCC (Figure 2D).

Additionally, we performed a polarization analysis of hepatic macrophages at the single-cell level. By scoring the expression of gene signatures associated with M1 (pro-inflammatory) and M2 (anti-inflammatory) phenotypes, we observed a gradual decline in M1 scores and a significant increase in M2 scores from cirrhosis to HCC. This dynamic shift suggests that, during the progression of chronic liver disease, macrophages transition from an immune-activated state focused on pathogen defense and inflammation toward an immunosuppressive phenotype involved in tissue remodeling and repair. Notably, macrophages in cirrhosis still maintain relatively high M1 activity, which is consistent with their role in sustaining inflammation and promoting fibrosis. However, in the HCC stage, M2-polarized macrophages become dominant, potentially facilitating angiogenesis, enhancing immune evasion, and promoting tumor-supportive remodeling of the microenvironment (Figure S2).

### 3.3. Communication Network Differences Between Cirrhosis and HCC

**Figure 3:**
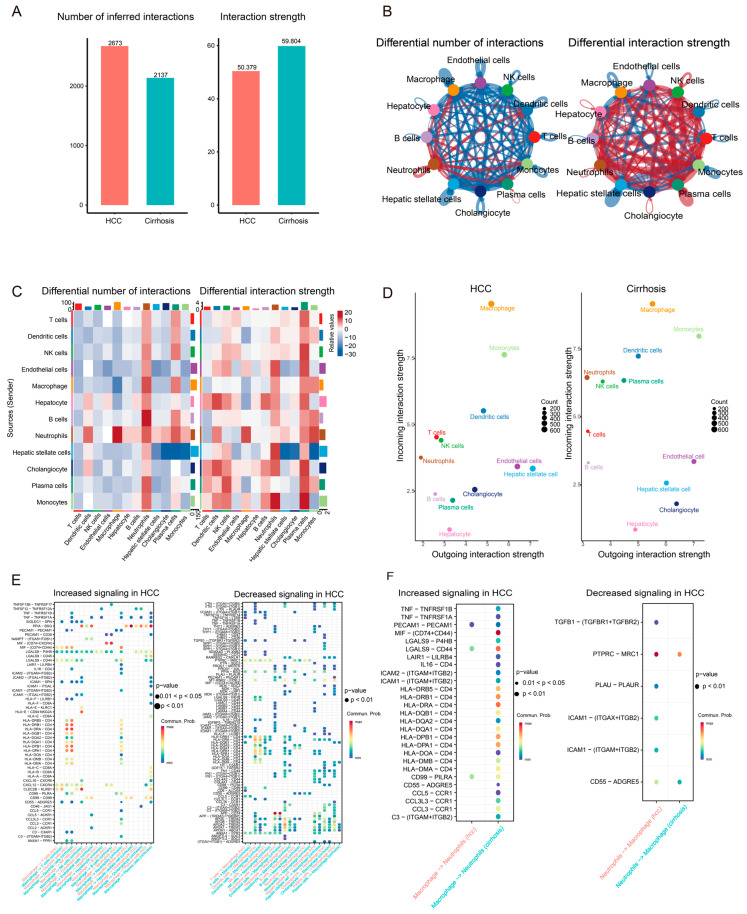
Intercellular communication landscape in cirrhosis and hepatocellular carcinoma (**A**) Overall comparison of the number and strength of cell–cell interactions between cirrhosis and HCC. (**B**) Differential interaction networks highlighting macrophage- and neutrophil-related communications; red edges indicate stronger interactions in cirrhosis, whereas blue edges indicate stronger interactions in HCC. (**C**) Cell type–specific differences in interaction number and strength; red represents higher values in cirrhosis, blue represents higher values in HCC. (**D**) Comparison of signaling strengths of major cell types as signal senders (outgoing) and receivers (incoming) in cirrhosis and HCC. (**E**) ligand–receptor signaling pathways, with inflammation- and chemotaxis-related pathways enriched in cirrhosis, and immunosuppressive or metabolic pathways predominant in HCC. (**F**) Differential communication between macrophages and neutrophils in cirrhosis and HCC.

To investigate the differences in intercellular communication between cirrhosis and HCC, we compared the overall cell–cell interaction networks in both conditions. We observed that despite the total number of interactions in HCC samples being significantly higher than in cirrhosis, the average strength of communication was relatively weaker. Notably, both the number and strength of communications involving macrophages and neutrophils were markedly higher in cirrhosis compared to HCC (Figure 3A,B). These results suggest that while HCC tissues harbor a more diverse array of cell types and broader intercellular connectivity, the signaling strength is weaker, reflecting a more immunosuppressive and functionally inert tumor microenvironment. In contrast, macrophages and neutrophils in cirrhotic tissue exhibit a more pro-inflammatory and active state, contributing robustly to immune surveillance and tissue repair processes.

Further analysis of macrophage-specific signaling revealed a consistently high incoming signaling strength in both cirrhosis and HCC, underscoring the central regulatory role of macrophages, which continuously receive external signals to modulate their functional states. Interestingly, the communication between macrophages and neutrophils was notably stronger during cirrhosis, indicating close interactions between these cells during active inflammation (Figure 3C,D).

We then analyzed ligand–receptor interactions to identify communication pathways that differ between cirrhosis and HCC. In cirrhotic tissues, several inflammatory and chemotactic ligand–receptor pairs, such as TNF–TNFRSF1A/B and MIF–CD74+CXCR4, were significantly more active than in HCC. Similarly, chemokine pathways including CCL5–CCR1 and CXCL12–CXCR4 were highly enriched. Multiple MHC class II–CD4 pairs (e.g., HLA-DRA–CD4, HLA-DRB1–CD4) also showed elevated expressions in cirrhosis. These findings indicate that cirrhosis is characterized by heightened inflammation-associated communication, with elevated expressions of pro-inflammatory ligands, chemokines, and antigen presentation molecules compared to HCC.

Additionally, ligand–receptor pairs such as SPP1–ITGAV, VTN–ITGAV, and HLA-related interactions were more active in cirrhosis, suggesting their potential roles in macrophage polarization, immune evasion, and tumor cell adhesion or migration. These interactions highlight the distinct functional states of macrophages in cirrhosis versus HCC (Figure 3E).

A focused analysis on the communication between macrophages and neutrophils revealed that macrophage-derived signaling to neutrophils was significantly enhanced in cirrhosis. This included pro-inflammatory pathways such as TNF–TNFRSF1A/B, MIF–CD74+CD44, and several MHC class II–CD4 interactions, suggesting that macrophages play an active antigen-presenting and inflammatory role during cirrhosis. Conversely, in HCC, these macrophage-to-neutrophil interactions were markedly reduced, and a subset of immunoregulatory signals—such as TGFB1–TGFBR1/2 and PTPRC–MRC1—mediated from neutrophils to macrophages became further restricted. This shift indicates the functional remodeling of macrophage–neutrophil communication during the transition from cirrhosis to HCC, reflecting the progression from an immune-activated state to one of immune suppression and evasion (Figure 3F).

### 3.4. Gene Selection by High-Dimensional Weighted Gene Co-Expression Network Analysis (hdWGCNA) and Gene Selection by Bulk RNA Sequencing and Lasso Regression

**Figure 4:**
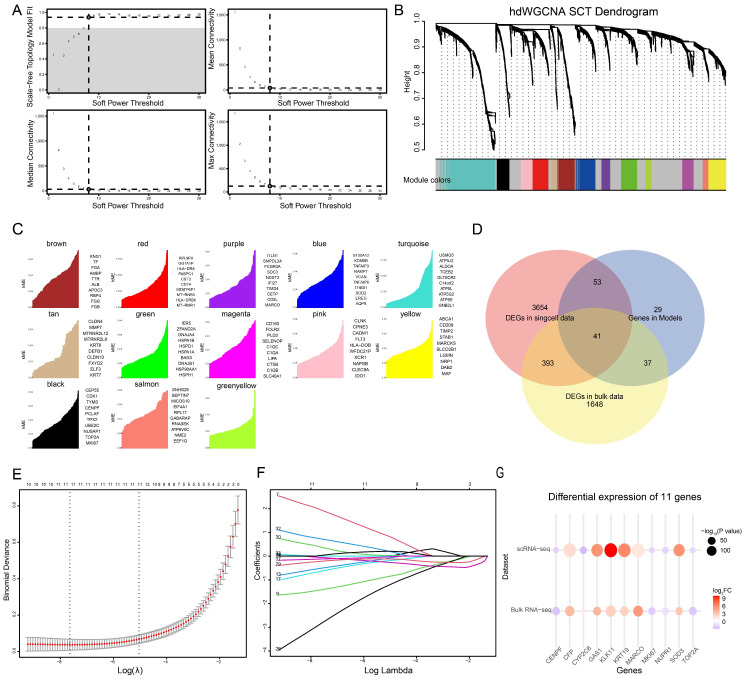
Identification of macrophage-associated gene modules and key diagnostic genes using hdWGCNA and Lasso regression. (**A**) Soft-thresholding power analysis for network topology, with power 8 selected as optimal. (**B**) Hierarchical clustering dendrogram of macrophage gene co-expression network, identifying 13 distinct gene modules. (**C**) Module membership plots showing top hub genes with highest intramodular connectivity. (**D**) Venn diagram showing the overlap among bulk RNA-seq DEGs, macrophage scRNA-seq DEGs, and hdWGCNA module genes, yielding 41 potential key genes. (**E**) Ten-fold cross-validation plot for Lasso regression to determine the optimal lambda value. (**F**) Lasso coefficient profiles and 11 genes selected for the final diagnostic model. (**G**) Comparative differential expression of 11 key genes between cirrhosis and HCC in bulk RNA-seq and single-cell RNA-seq datasets.

To identify gene modules associated with cirrhosis and HCC, we extracted all cells annotated as macrophages from the single-cell RNA sequencing data and performed hdWGCNA following appropriate preprocessing. After constructing meta cells based on the macrophage expression profiles from cirrhotic and HCC tissues, we scanned across a range of soft-thresholding powers, selecting a power of eight as optimal (Figure 4A). Using this threshold, a gene co-expression network was constructed, and hierarchical clustering analysis was performed, resulting in the identification of 13 distinct gene modules (Figure 4B).

We quantified gene connectivity within each non-gray module to evaluate the centrality of genes and identified the top 20 hub genes per module. These hub genes represent the most interconnected members of their respective modules and are likely to play key roles in relevant biological processes (Figure 4C). To further narrow down functional modules associated with disease progression, we compared the differentially expressed genes (DEGs) between cirrhotic and HCC macrophages with the identified module-specific hub genes. Modules with a notable overlap were selected, and their corresponding genes were used for downstream analysis.

To identify candidate genes with high diagnostic value from the module-associated genes, we first analyzed the bulk RNA sequencing dataset GSE25097. Differential expression analysis was performed, and 2119 significantly differentially expressed genes (DEGs) were identified based on an adjusted *p*-value < 0.01 and an absolute log2 fold change (|log2FC|) > 1. We then intersected these DEGs with both the macrophage-related DEGs identified from single-cell RNA sequencing and the module genes derived from hdWGCNA. This resulted in a set of 41 overlapping and potentially key genes (Figure 4D).

Subsequently, we used the GSE25097 dataset as the training cohort to construct a least absolute shrinkage and selection operator (Lasso) regression model. Ten-fold cross-validation was performed using the R package glmnet to determine the optimal lambda value, which was identified as 0.0004814197 (Figure 4E). The final model included 11 selected genes: CENPF, CFP, CYP2C8, GAS1, KLK11, KRT19, MARCO, MKI67, NUPR1, SOD3, and TOP2A (Figure 4F). The differential expression patterns of these genes in the training cohort were largely consistent with those observed in the single-cell RNA sequencing dataset (Figure 4G), with the exception of CYP2C8, which exhibited an opposite expression trend between the two datasets. Specifically, KLK11, MARCO, CFP, KRT19, GAS1, SOD3, and CYP2C8 were downregulated in HCC. These genes are functionally associated with tumor suppression, the induction of apoptosis, and the classification of HCC molecular subtypes; their reduced expression may contribute to unfavorable prognosis in HCC patients [30,31,32,33,34]. In contrast, TOP2A, CENPF, MKI67, and NUPR1 were upregulated in HCC. These genes are primarily involved in cell cycle regulation, particularly G2/M phase transition during mitosis, as well as cell proliferation and macrophage M2 polarization; thus, their overexpression is also strongly associated with poor clinical outcomes [35,36,37,38]. Therefore, it is biologically plausible and clinically reasonable to consider these genes as potential diagnostic biomarkers for HCC.

### 3.5. Model Construction and Performance Evaluation

In this study, we selected 11 key genes to construct three diagnostic models: a least absolute shrinkage and selection operator (Lasso) regression model, a random forest model, and an extreme gradient boosting (XGBoost) model. The training dataset was derived from GSE25097, which included 308 samples (40 cirrhosis and 268 hepatocellular carcinoma samples). The independent validation dataset was obtained from GSE14323, containing 96 samples (41 cirrhosis and 55 HCC samples). These two datasets were subsequently merged to form a unified cohort for further evaluation.

**Figure 5:**
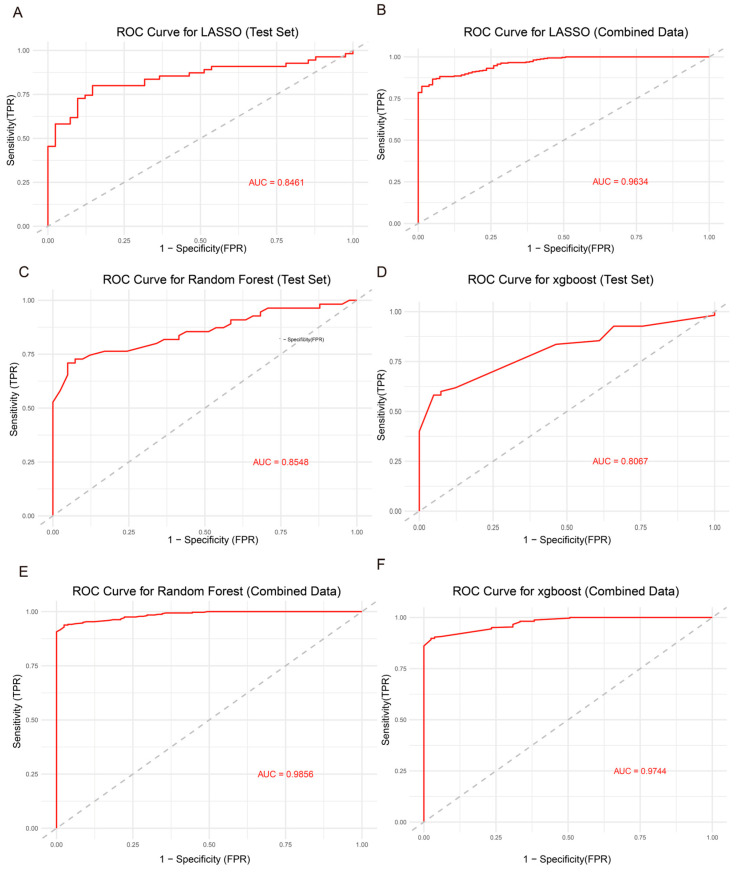
Diagnostic performance of Lasso, random forest, and XGBoost models in distinguishing cirrhosis from HCC. (**A**) ROC curve of the Lasso regression model on the independent validation dataset. (**B**) ROC curve of the Lasso model on the combined dataset of training and validation samples. (**C**) ROC curve of the random forest model on the independent validation dataset. (**D**) ROC curve of the XGBoost model on the independent validation dataset. (**E**) ROC curve of the random forest model on the combined dataset. (**F**) ROC curve of the XGBoost model on the combined dataset.

Model performance was assessed using the area under the receiver operating characteristic curve (AUC). The Lasso regression model demonstrated good discriminatory power on the independent test set, with an AUC of 0.8461 (Figure 5A). After integrating the training and test datasets, the AUC increased to 0.9634 (Figure 5B), indicating excellent predictive performance.

The random forest and XGBoost models also performed well on the independent validation dataset, producing AUCs of 0.8548 and 0.8067 (Figure 5C,D), respectively. When evaluated on the combined dataset, the AUCs increased to 0.9856 and 0.9744 (Figure 5E,F), further confirming the robustness and diagnostic potential of these models in distinguishing between cirrhosis and HCC.

To further evaluate the clinical applicability of the 11-gene signature, optimal cutoff values were determined for each model using ROC curve analysis. These cutoffs allowed patient stratification into high-risk (positive) and low-risk (negative) groups. The LASSO, XGBoost, and Random Forest models achieved robust diagnostic performance, with sensitivity and specificity values ranging from 0.78 to 0.95 across independent test datasets (Table S2). In addition, the importance of each gene in the different models was assessed and visualized (Figure S3), highlighting their relative contributions to the predictive performance. This demonstrates that the expression patterns of the selected genes can be directly applied in clinical settings to distinguish cirrhosis patients at high risk of HCC from those at lower risk. In clinical practice, the expression levels of these genes can be measured in patient samples and input into the predictive models to determine the risk category based on the established cutoff, thereby guiding early monitoring and intervention.

## 4. Discussion

Hepatocellular carcinoma is a globally prevalent malignancy and ranks as the third leading cause of cancer-related mortality worldwide [39,40]. Liver cirrhosis, frequently resulting from chronic liver diseases such as hepatitis B virus infection, is a major risk factor for HCC and represents a common pathological transition toward tumorigenesis [41,42]. Elucidating the cellular and molecular alterations that occur during the progression from cirrhosis to HCC is crucial for improving early diagnostic strategies and patient outcomes. In this study, we systematically analyzed single-cell transcriptomic data from patients with HBV infection, cirrhosis, and HCC, focusing on disease-associated changes in the hepatic microenvironment. Cell annotation and compositional analysis revealed a marked increase in macrophage abundance across disease stages, suggesting the potential key role of macrophages in the transition from cirrhosis to HCC. Unlike previous hypothesis-driven studies, we employed a data-driven approach to identify significantly altered cell types and subsequently focused on macrophages for in-depth characterization of their transcriptional and functional states in cirrhosis and HCC. Through differential gene expression analysis, M1/M2 polarization scoring [43], and intercellular communication profiling, we comprehensively identified the phenotypic differences in macrophages between these two disease states. Furthermore, we applied high-dimensional weighted gene co-expression network analysis to construct macrophage-specific co-expression modules, identifying functionally relevant gene clusters and hub genes. These findings were cross-validated with bulk RNA-seq data to enhance the robustness and clinical relevance of our results. Finally, using LASSO regression, we identified 11 key genes and developed predictive models based on multiple machine learning algorithms. These models demonstrated excellent diagnostic performance with area under the curve values exceeding 0.9 across independent datasets, underscoring their generalizability and translational potential. Collectively, our findings reveal dynamic transcriptional changes in macrophages during the cirrhosis-to-HCC transition, identifying novel biomarker candidates and analytic frameworks for early HCC diagnosis. Importantly, the final 11 genes were prioritized because they consistently showed stable predictive performance across single-cell and bulk datasets, were repeatedly validated by multiple machine learning approaches, and exhibited clear biological relevance to macrophage function and hepatocarcinogenesis, whereas other candidates lacked such robustness.

It is noteworthy that although approximately 80% of HCC arises in the context of cirrhosis, a subset of patients develop HCC without cirrhosis through distinct mechanisms. Metabolism-related single nucleotide polymorphisms (such as PNPLA3, TM6SF2, and HSD17B13) increase susceptibility to HCC in non-cirrhotic individuals, while environmental carcinogens, including aflatoxin B1 and aristolochic acid, can directly induce somatic mutations and synergize with HBV infection to promote tumorigenesis. At the molecular level, TERT promoter mutations represent the most frequent early “gatekeeper” event, with additional alterations in CTNNB1, TP53, and epigenetic regulators (BAP1, ARID1A/B, ARID2) further driving tumor progression. Importantly, these alterations are not dependent on a fibrotic background, providing a plausible explanation for HCC development in non-cirrhotic livers. Moreover, in NASH-related HCC, immunological mechanisms play a critical role, as autoreactive CD8+PD1+ T cells induce hepatocyte death and impair immune surveillance, thereby facilitating tumor formation.

Liver fibrosis is characterized by the excessive accumulation of extracellular matrix (ECM) components in the liver due to chronic liver injury from various etiologies. By contrast, cirrhosis represents an advanced stage of fibrosis [44]. In our study, the proportion of macrophages increased progressively with disease progression, reflecting their dynamic functional states in different pathological contexts. Notably, fibrosis-associated pathways were highly enriched in macrophages from cirrhotic tissues, including extracellular matrix organization, extracellular structure organization, and pathways related to chemotaxis and leukocyte migration, which are directly involved in ECM deposition and macrophage recruitment. This phenomenon is consistent with the pathological mechanisms of liver fibrosis, in which damaged or apoptotic hepatocytes release damage-associated molecular patterns (DAMPs) that activate hepatic stellate cells (HSCs), promoting their transdifferentiation into collagen-producing myofibroblasts [41,45]. During this process, lymphocytes and macrophages are recruited and activated, secreting pro-inflammatory and pro-fibrotic cytokines that further stimulate activation of HSC and progression of fibrosis [46,47]. The activated HSCs, in turn, contribute to ECM production as part of tissue repair responses [48]; however, persistent ECM accumulation eventually leads to pathological fibrosis. In our study, hepatic stellate cells were also identified, supporting the presence of fibrogenic activity and further validating the functional phenotype of macrophages observed in cirrhosis.

In contrast, macrophages in hepatocellular carcinoma (HCC) exhibited reduced enrichment of immune-related pathways, such as leukocyte-mediated immunity, lymphocyte-mediated immunity, and antigen receptor-mediated signaling; however, they showed increased activity in pathways associated with metabolic reprogramming. Tumor cells in HCC frequently face hypoxic and nutrient-deprived conditions and undergo adaptive metabolic shifts—referred to as metabolic reprogramming—to meet high energy demands or reshape the tumor microenvironment in favor of growth [49]. Pathways such as amino acid metabolism, fatty acid metabolism, and organic acid catabolism observed in this study are strongly associated with the Warburg effect and alter amino acid metabolism, both of which are hallmarks of tumor metabolic reprogramming [50,51]. The weakened immune response to HCC may be largely attributed to the immunosuppressive roles of tumor-associated macrophages (TAMs) [52]. M2-polarized TAMs can directly interact with myeloid-derived suppressor cells (MDSCs) and actively suppress T-cell-mediated anti-tumor immunity [53]. Moreover, they express immune-inhibitory surface molecules, such as PD-L1 and B7-H4 [54], which contribute to immune evasion. Collectively, these findings explain why immune activation in HCC is significantly lower compared to cirrhosis.

In recent years, a growing body of research in the literature has highlighted the pivotal role of macrophage polarization in numerous pathophysiological processes, including inflammation, tumor progression, tissue repair, and metabolic regulation [55,56]. Interestingly, these pathological mechanisms are also prominent features of liver diseases, suggesting a close association between macrophage polarization and the progression or resolution of hepatic disorders such as hepatitis, fibrosis, and hepatocellular carcinoma (HCC) [57,58]. In general, macrophage polarization is broadly classified into two phenotypes: classically activated M1 and alternatively activated M2 macrophages [43]. M1 macrophages are primarily involved in antigen presentation and exhibit pro-inflammatory, antimicrobial, and anti-tumor functions. In contrast, M2 macrophages are associated with anti-inflammatory responses, tissue remodeling, parasite resistance, and the promotion of angiogenesis, immune regulation, and tumor progression [59]. In this study, we quantified macrophage polarization states across hepatitis, cirrhosis, and HCC samples. We observed a progressive decline in M1 polarization scores and a corresponding increase in M2 polarization scores as disease advanced. Chronic hepatitis induced by viral infections, including hepatitis B virus (HBV) or hepatitis C virus (HCV), shows enhanced M1 polarization, while M2 polarization is suppressed [60,61,62]. In contrast, the M2 polarization of macrophages in HCC plays a key role in promoting immune suppression, angiogenesis, and cancer cell metastasis, resulting in the highest M2 polarization scores observed in HCC tissues. These findings are highly consistent with previous studies and further reinforce the role of M2-type macrophages in shaping an immunosuppressive tumor microenvironment that favors HCC progression [48].

In the cell–cell communication analysis of this study, we observed that although the overall number of intercellular interactions in cirrhotic tissues was lower than that in hepatocellular carcinoma (HCC), the communication frequency of T cells and neutrophils was notably higher in cirrhosis. This may be attributed to the immunosuppressive microenvironment and metabolic reprogramming characteristic of HCC, consistent with our pathway enrichment analysis findings. Furthermore, macrophage–neutrophil interactions were significantly enhanced in cirrhotic tissues, particularly in signaling pathways such as TNF–TNFRSF1A/B, MIF–(CD74+CD44), and multiple MHC class II ligand–receptor pairs. These results suggest that macrophages may actively participate in pro-inflammatory signaling and antigen presentation during the early stages of liver disease. In contrast, these pathways were generally weakened in HCC, and several immunoregulatory signals, including TGFB1–TGFBR and PTPRC–MRC1, were also significantly downregulated, reflecting the formation of an immunosuppressive or immune-evasive environment. This shift in communication patterns may contribute to the transition from chronic inflammation to tumorigenesis, supporting the concept of the “inflammation-to-cancer continuum”.

Traditional weighted gene co-expression network analysis (WGCNA) is primarily designed for bulk transcriptomic data and may perform suboptimally in high-dimensional scRNA-seq datasets. To address this, we applied hdWGCNA to identify co-expression modules from macrophage-specific scRNA-seq data. Modules with a significant overlap of hub genes and macrophage-specific differentially expressed genes were selected for further analysis. Considering that macrophage-derived feature genes might not show significant differential expression at the bulk tissue level, we performed differential expression analysis using bulk transcriptomic datasets of liver cirrhosis and HCC. We then filtered genes that were significantly altered and exhibited consistent expression trends with macrophage-specific genes. Eleven genes were identified, many of which have been previously reported as biomarkers or functional regulators in HCC or other cancers. CENPF serves as both an important prognostic biomarker and a key oncogenic driver in HCC; its role has been independently confirmed through both bioinformatics and experimental validation, and it can cooperate with FOXM1 to co-regulate downstream targets such as POLD1, thereby promoting hepatocarcinogenesis and tumor progression [36,63]. CFP is significantly downregulated in HCC, and its low expression is associated with poorer overall survival (OS) and disease-free survival (DFS), suggesting that it may serve as a potential prognostic biomarker and immune-related therapeutic target [64]. CYP2C8 is also downregulated in HCC tissues, and its reduced expression correlates with unfavorable OS and DFS. Moreover, CYP2C8, together with FCN3 and other prognosis-related genes, was incorporated into an 11-gene risk prediction model that accurately predicted overall survival [64,65,66]. KRT19 interacts with long non-coding RNAs to activate signaling pathways related to tumor proliferation and metastasis [67,68]. MARCO is downregulated in HCC tissues, and its low expression is associated with higher alpha-fetoprotein (AFP) levels, microvascular invasion, and poorer OS and DFS following liver transplantation. MARCO has been identified as an independent prognostic factor and is mainly expressed in macrophages, where it is linked to M2-like polarization [69]. MKI67 is an important prognostic gene in HCC, showing high expression in HBV/HCV-associated cases and being incorporated into hypoxia-related prognostic models, indicating its involvement in virus-induced tumor progression and hypoxia-driven cellular proliferation [37,70]. NUPR1 is highly expressed in tumor-associated macrophages within HCC and is related to immunosuppressive polarization and poor immunotherapeutic response, suggesting its potential as a biomarker and therapeutic target in HCC [71]. SOD3 is downregulated in liver fibrosis, and its deficiency promotes hepatic stellate cell activation and epithelial–mesenchymal transition (EMT), thereby aggravating liver injury and collagen deposition [72]. Finally, TOP2A is significantly upregulated in HCC tissues, and its high expression is associated with advanced tumor stage, poor differentiation, and unfavorable prognosis, particularly in Asian populations, suggesting that TOP2A may serve as a molecular marker for HCC progression and prognosis [35,73].These genes are closely linked to pathways involved in cirrhosis-to-HCC progression and patient prognosis, making them suitable for diagnostic modeling. Using these genes, we constructed machine learning models—including LASSO regression, random forest, and XGBoost—trained on bulk transcriptomic data and validated on independent cohorts. All models demonstrated robust performance in distinguishing cirrhosis from HCC, underscoring their potential clinical utility. In this study, we observed that some genes showed opposite expression trends between single-cell RNA sequencing (scRNA-seq) and bulk RNA sequencing (bulk RNA-seq). This discrepancy largely reflects the distinct nature of the two technologies: scRNA-seq highlights features of minor cell subpopulations, whereas bulk data represent averaged tissue signals and are strongly influenced by changes in cell composition. For instance, CYP2C8 appeared downregulated in bulk HCC data but retained expression in specific subsets at the single-cell level, suggesting regulation shaped by both cellular composition and functional states. Such differences are not contradictory but rather underscore the complementary value of integrating scRNA-seq and bulk RNA-seq for a more comprehensive understanding of disease progression.

Despite the novel insights provided by this study, several limitations should be acknowledged. First, our analyses were primarily based on publicly available single-cell RNA sequencing (scRNA-seq) and bulk RNA sequencing datasets derived from different cohorts. The inherent batch effects and population heterogeneity may limit the generalizability of our findings. Moreover, the current conclusions are mainly supported by bioinformatic analyses without experimental validation, which remains a key limitation. In future work, we plan to perform quantitative real-time PCR (qRT-PCR) and immunohistochemical (IHC) assays using cirrhosis and HCC samples to validate the expression and biological relevance of the 11 macrophage-related genes identified in this study.

Second, although the machine learning–based diagnostic models demonstrated promising performance, their robustness requires further evaluation in larger, multi-center independent cohorts. Future studies should validate the 11 macrophage-related genes and predictive models in larger, multi-center cohorts and incorporate longitudinal data to confirm their predictive value. Mechanistic experiments are also required to clarify how these genes regulate macrophage remodeling and the tumor microenvironment. Advances in spatial transcriptomics and multi-omics integration will further provide a comprehensive biological framework and accelerate their translation into personalized clinical applications.

## Data Availability

The data presented in this study are available in the Gene Expression Omnibus (GEO) database at https://www.ncbi.nlm.nih.gov/geo/, accessed on 30 September 2024, reference numbers GSE136103, GSE186343, GSE242889, GSE202642, GSE25097, and GSE14323. These data were derived from the following resources available in the public domain: https://www.ncbi.nlm.nih.gov/geo/query/acc.cgi?acc=GSE136103, https://www.ncbi.nlm.nih.gov/geo/query/acc.cgi?acc=GSE186343, https://www.ncbi.nlm.nih.gov/geo/query/acc.cgi?acc=GSE242889, https://www.ncbi.nlm.nih.gov/geo/query/acc.cgi?acc=GSE202642, https://www.ncbi.nlm.nih.gov/geo/query/acc.cgi?acc=GSE25097, and https://www.ncbi.nlm.nih.gov/geo/query/acc.cgi?acc=GSE14323 (accessed on 30 September 2024).

## Supplementary Materials

The following supporting information can be downloaded at: https://www.mdpi.com/article/10.3390/genes16101213/s1, Figure S1: UMAP plots showing cell annotations for integrated and disease-specific datasets. Figure S2: Polarization states of macrophages in hepatitis, cirrhosis, and HCC. Figure S3: Feature importance of LASSO, XGBoost, and Random Forest models; Table S1: Summary of cell numbers for each sample and total cells in each disease group; Table S2: Summary of performance metrics (AUC, sensitivity, specificity) for predictive models.
