# Supplementary material for: Integrative Single-Cell and Bulk Transcriptomic Analysis Identifies Macrophage-Related Gene Signatures Predictive of Hepatocellular Carcinoma in Cirrhosis
Source: Genes (Basel). 2025 Oct 15;16(10):1213. doi: 10.3390/genes16101213 (PMC12562699; doi:10.3390/genes16101213)
Supplement: Supplementary file 1 [file genes-16-01213-s001.zip › genes-3830310-supplementary.pdf]

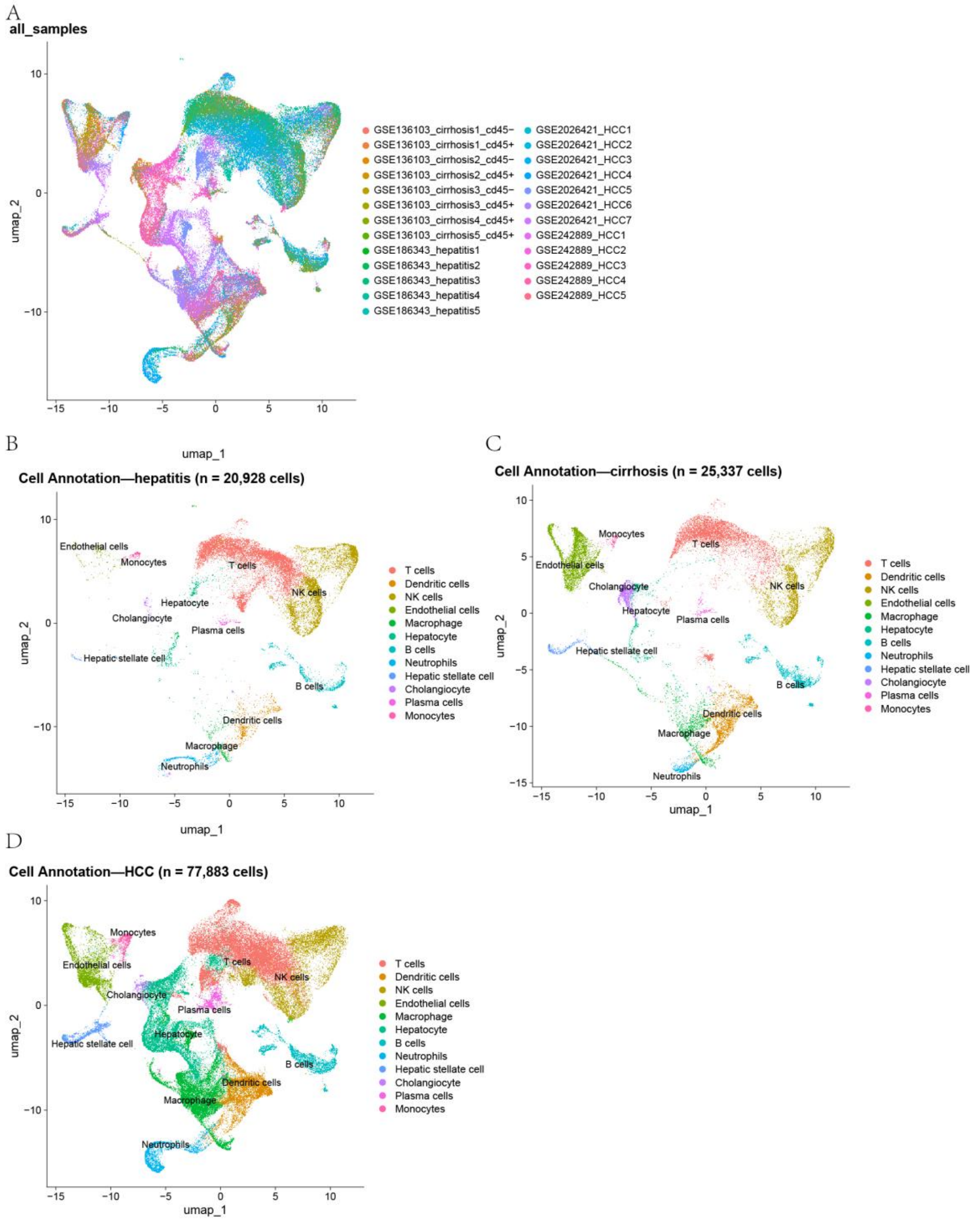

Figure S1. UMAP plots showing cell annotations for integrated and disease-specific datasets. (A) UMAP of all integrated samples colored by dataset ID (to evaluate batch effects） (B) Cell annotation of hepatitis samples (n = 20,928). (C) Cell annotation of cirrhosis samples (n = 25,337). (D) Cell annotation of HCC samples (n = 77,883).

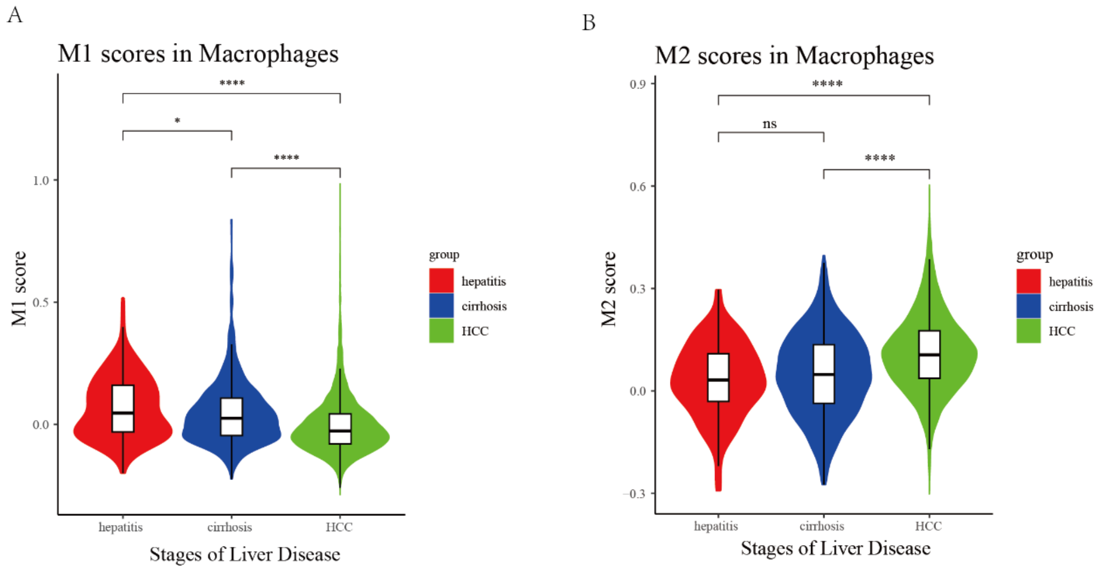

Figure S2. Polarization states of macrophages in hepatitis, cirrhosis, and HCC. (A) M1 polarization scores of macrophages across hepatitis, cirrhosis, and HCC. (B) M2 polarization scores of macrophages across hepatitis, cirrhosis, and HCC.

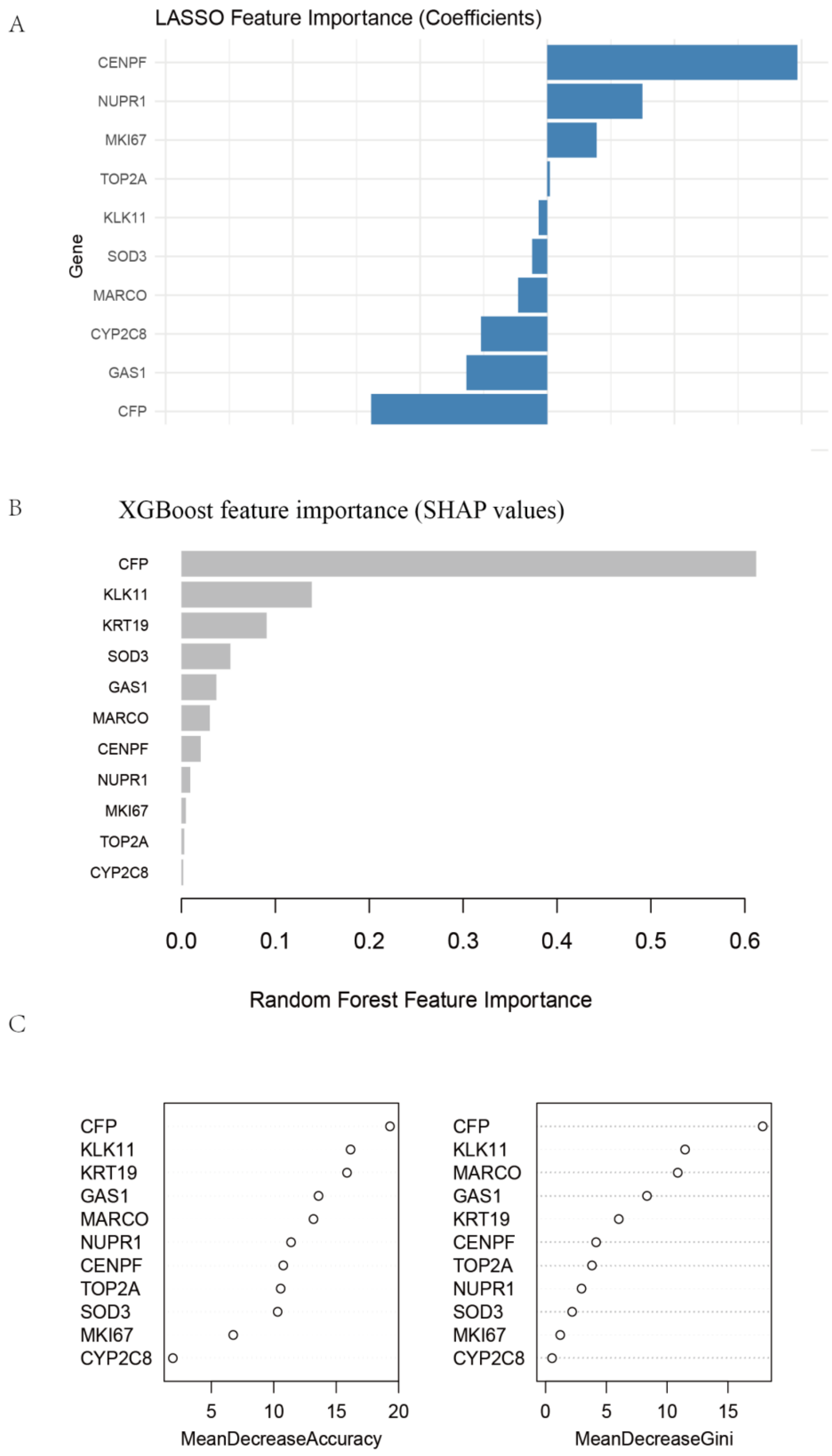

=

Figure S3. Feature importance of LASSO, XGBoost, and Random Forest models (A) LASSO feature importance (Coefficients). (B) XGBoost feature importance (SHAP values). (C) Random Forest feature importance.

Table S1. Summary of cell numbers for each sample and total cells in each disease group

**Disease**	**Sample ID**	**Number of cells**
Cirrhosis	GSE136103_cirrhosis1_cd45-	3295
Cirrhosis	GSE136103_cirrhosis1_cd45+	2243
Cirrhosis	GSE136103_cirrhosis2_cd45-	3489
Cirrhosis	GSE136103_cirrhosis2_cd45+	3267
Cirrhosis	GSE136103_cirrhosis3_cd45-	3499
Cirrhosis	GSE136103_cirrhosis3_cd45+	1863
Cirrhosis	GSE136103_cirrhosis4_cd45+	4794
Cirrhosis	GSE136103_cirrhosis5_cd45+	2887
Cirrhosis total	—	25,337
Hepatitis	GSE186343_hepatitis1	1308
Hepatitis	GSE186343_hepatitis2	4669
Hepatitis	GSE186343_hepatitis3	3678
Hepatitis	GSE186343_hepatitis4	5198
Hepatitis	GSE186343_hepatitis5	6075
Hepatitis total	—	20,928
HCC	GSE2026421_HCC1	9925
HCC	GSE2026421_HCC2	10325
HCC	GSE2026421_HCC3	3933
HCC	GSE2026421_HCC4	6132
HCC	GSE2026421_HCC5	6711
HCC	GSE2026421_HCC6	6399
HCC	GSE2026421_HCC7	12088
HCC	GSE242889_HCC1	4551
HCC	GSE242889_HCC2	4384
HCC	GSE242889_HCC3	3742
HCC	GSE242889_HCC4	4266
HCC	GSE242889_HCC5	5427
HCC total	—	77,883
Disease	Sample ID	Number of cells
Cirrhosis	GSE136103_cirrhosis1_cd45-	3295
Cirrhosis	GSE136103_cirrhosis1_cd45+	2243
Cirrhosis	GSE136103_cirrhosis2_cd45-	3489
Cirrhosis	GSE136103_cirrhosis2_cd45+	3267
Cirrhosis	GSE136103_cirrhosis3_cd45-	3499
Cirrhosis	GSE136103_cirrhosis3_cd45+	1863
Cirrhosis	GSE136103_cirrhosis4_cd45+	4794
Cirrhosis	GSE136103_cirrhosis5_cd45+	2887

Table S2. Summary of performance metrics (AUC, sensitivity, specificity) for predictive models

Model	Cutoff	AUC	Sensitivity	Specificity
LASSO	0.83	0.843	0.782	0.780
XGBoost	0.89	0.807	0.618	0.878
Random Forest	0.82	0.850	0.745	0.780
